# Boosting Chocolate Nutrition with Sous Vide-Processed White Champignon (*Agaricus bisporus*) Powder: A Functional and Sustainable Approach

**DOI:** 10.3390/foods14223808

**Published:** 2025-11-07

**Authors:** Szintia Jevcsák, Gréta Törős, Gerda Diósi, Xhensila Llanaj, József Prokisch

**Affiliations:** 1 Institute of Food Technology, Faculty of Agricultural and Food Sciences and Environmental Management, University of Debrecen, Böszörményi Str. 138, H-4032 Debrecen, Hungary; diosi@agr.unideb.hu; 2 Department of Animal Husbandry, Institute of Animal Science, Biotechnology and Nature Conservation, Faculty of Agricultural and Food Sciences and Environmental Management, University of Debrecen, Böszörményi Str. 138, H-4032 Debrecen, Hungaryjprokisch@agr.unideb.hu (J.P.); 3 Doctoral School of Food Science, University of Debrecen, Böszörményi Str. 138, H-4032 Debrecen, Hungary

**Keywords:** chocolate, *Agaricus bisporus*, sous-vide processing, functional food, nutritional enhancement, fortified chocolate, functional confectionery, dietary fiber enrichment, sensory evaluation

## Abstract

With growing demand for functional foods, mushroom-based ingredients are gaining popularity. The typical white mushroom (*Agaricus bisporus*) is particularly valued for its bioactive compounds and shows promise as a nutritional enhancer in widely consumed products, such as chocolate. This study examined the fortification of dark, milk, and white chocolates with freeze-dried, sous-vide processed *A. bisporus* powder at 4%, 6%, and 8% levels. Analyses focused on protein content, dietary fiber, essential minerals, texture, and sensory characteristics. Mushroom addition notably improved nutritional values. In white chocolate, protein increased from 6.04% to 8.92%, while dark chocolate with 8% fortification reached 13.25%, compared to 11.09% in the control. The magnesium content also increased significantly, from 2579 mg/kg to 3184 mg/kg. Total dietary fiber also showed a significant improvement. Texture analysis revealed a reduction in firmness, with the 8% *A. bisporus* powder fortified dark chocolate formulation softening from 24,685 g·s to 10,633 g·s. Despite these changes, sensory evaluation confirmed that taste and appearance remained acceptable. Overall, incorporating *A. bisporus* powder into chocolate improved its nutritional profile while introducing moderate changes to texture. These findings highlight its potential as a functional ingredient in the development of healthier confectionery products.

## 1. Introduction

Theobroma—Food of the Gods: Chocolate derives from the cocoa bean of *Theobroma cacao* L., a member of the Sterculiaceae family. Four main cocoa types are recognized: Criollo (≈5% of global production), Forastero (smaller, purple beans), Nacional (delicate flavor), and Trinitario (a hybrid of Criollo and Forastero) [1,2]. Cocoa is a key agricultural export, underpinning the economies of many nations in West Africa, Southeast Asia, and South America [3]. Over 70% of global cocoa originates in West Africa [4].

Milk chocolate leads in consumer preference across regions, though dark chocolate especially with high cocoa content offers greater polyphenol levels and associated health benefits [5]. Over time, cocoa and chocolate have been celebrated for their potential nutritional and medicinal benefits. Studies have linked them to reduced risks of cardiovascular disease, cancer, and other chronic conditions [6,7]. Chocolate also contributes to the intake of macronutrients (carbohydrates, protein, and fats) and minerals such as magnesium, potassium, copper, and iron [8,9]. Moderate consumption, typically defined as 20–40 g per day of dark chocolate (≥70% cocoa), has been associated with beneficial cardiovascular and metabolic effects [10,11]. Dark chocolate with a cocoa dry matter content of more than 60% can also serve as a source of serotonin [12]. Cocoa products are rich in polyphenols and have antioxidant, antiviral, anti-inflammatory, anticarcinogenic, and cholesterol-modulating properties [13,14]. However, not all chocolate is equally nutritious, since composition varies by cocoa content, milk addition, and other ingredients [5,15]. Modern consumers increasingly seek products that offer both indulgence and health benefits [16].

Mushrooms are nutrient-dense foods rich in dietary fiber, protein, minerals, and moderate complex carbohydrates. Their low-glycemic-index carbohydrates help regulate blood sugar. Protein content ranges widely, from 12% to 38% depending on species [17] and typically includes all essential amino acids [18]. They also provide essential minerals such as potassium, phosphorus, calcium, zinc, and iron [19]. The champignon mushroom (*A. bisporus*) is among the most widely cultivated and consumed species worldwide [20]. Its medicinal value stems from bioactive compounds present in both its mycelium and fruiting body [21], conferring antioxidant, antimicrobial, and anticancer activities [22,23,24]. It is particularly rich in polyphenols, ergothioneine [25,26], as well as polysaccharides such as β-glucan and chitosan [27,28,29], and antimicrobial peptides [30]. These constituents support gut health by modulating the microbiota and may reduce the risk of disease [31]. Moreover, mushroom cultivation is considered an environmentally sustainable practice, requiring relatively modest inputs of land, energy, and water compared to animal-based protein sources [32]. The valorization of mushroom powders and byproducts further supports circular economy approaches, reducing food waste [33].

Transforming mushrooms into appealing, value-added foods, such as nutritious chocolates, is a promising approach, especially given children’s affinity for chocolate [34]. Given that many people do not meet the recommended fiber intake (21–40 g/day, depending on age, sex, and health) [35], adding dehydrated mushroom powder to chocolate can boost both fiber and protein while enhancing antioxidant capacity [36,37]. Moreover, chocolate is theorized to promote the release of endorphins [38]. Enriching chocolate with mushroom powder may thus offer a tasty route to health promotion for both children and adults.

This study aimed to develop and evaluate chocolate formulations enriched with white button mushroom powder produced through the combination of sous vide and freeze-drying techniques. The research focused on assessing nutritional enhancement (protein, fiber, minerals), functional and sensory properties, textural changes, and the sustainability perspective of mushroom-based fortification. By combining culinary appeal with health-oriented innovation, this work contributes to the growing field of functional confectionery. It highlights the potential of mushrooms as sustainable ingredients in modern food systems.

## 2. Materials and Methods

### 2.1. Experimental Design

**Figure 1:**
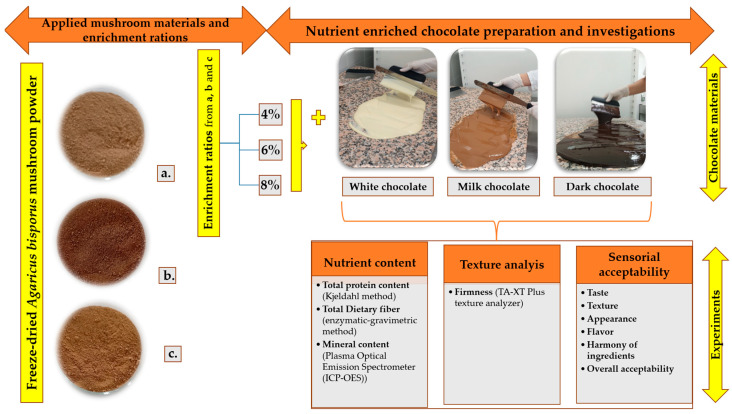
The conception of the study with the applied samples (a. I. Quality; b. II. Quality, and c. Above II. Quality of cooked and freeze-dried *A. bisporus* mushroom powder as seen in the picture), used concentration (4, 6, and 8%), and applied methods for investigating the nutrient-rich chocolates.

This experiment was designed to investigate the feasibility of incorporating white button mushroom (*Agaricus bisporus*) powder into chocolate to enhance its nutritional profile while maintaining consumer acceptability. The experimental design consisted of producing three quality grades of mushroom powder, applying them to three types of chocolate bases (dark, milk, and white), and enriching each with three concentrations (4%, 6%, and 8%). This research design allowed for the assessment of multiple variables, including nutrient content (protein, fiber, minerals), textural changes, and sensory responses. Fresh mushrooms were processed and analyzed at the Nanofood Laboratory, Institute of Animal Science, Biotechnology, and Nature Conservation. Chocolate sample production was performed at the Food Innovation Center, University of Debrecen. Figure 1 illustrates the overall conception of the study, including sample types and key analytical methods.

### 2.2. Mushroom Powder Processing and Measurements

#### 2.2.1. Sous-Vide Treatment of Fresh Mushrooms

Fresh Mushroom Processing Method: Freshly harvested *A. bisporus* mushrooms, classified into three distinct quality grades, were procured in 2023 from Magyar Gomba Kertész Kft. (Demjén, Hungary). The mushrooms were meticulously washed under warm running water and cut into smaller pieces to facilitate uniform cooking. The prepared mushroom pieces were then vacuum-sealed and cooked using sous vide technology and equipment supplied by VWR International Hungary Kft (Debrecen, Hungary).

This involved cooking at 90 °C for 4 h, allowing for a thorough study of the effects of sous vide cooking on the mushrooms’ nutritional properties. Among the options, the 90 °C for 4 h setting stood out, delivering the best results in preserving the mushrooms’ structure, color, and flavor. This treatment aligns with findings in previous studies, which highlight its effectiveness in deactivating enzymes and ensuring microbial safety under similar processing conditions [39,40,41].

#### 2.2.2. Freeze-Drying of Cooked Mushroom Samples

Cooked Mushroom Preservation Method: The mushrooms were subjected to lyophilization following the sous vide cooking process. This freeze-drying was carried out over 24 h using a freeze dryer (VWR International Hungary) set at −80 °C with a vacuum pressure of 0.04 mbar. After freeze-drying, the mushrooms were finely ground into a powder using a colloid mill. This process preserved the mushrooms’ flavor and nutritional content, converting them into a versatile powdered form. The resulting mushroom powders were then utilized to fortify chocolate, showcasing their potential in various culinary and industrial applications.

#### 2.2.3. Nutritional Characterization of Mushroom Powder

The total protein content of the mushroom powder was quantified using the Kjeldahl method. The results indicated that I. quality samples had a protein content of 28.15 ± 4.17%, II. quality samples contained 24.05 ± 12.80%, and above II. quality samples exhibited the highest protein content at 40.68 ± 0.21%. Dietary fiber analysis was performed using the enzymatic gravimetric method as outlined in Directive 3-2-2008/1. The I. quality mushroom powder contained 47.62 ± 0.22% dietary fiber, II. quality samples had a significantly higher fiber content of 54.89 ± 0.14%, while above II. The quality samples exhibited a fiber content of 47.08 ± 0.71%.

Elemental analysis was conducted using an iCAP 6300 Dual Plasma Optical Emission Spectrometer (ICP-OES) (Termofischer Scientific, Cambridge, UK). The I. quality mushroom powder demonstrated higher concentrations of key elements than the II. quality mushroom powder. Quality mushroom powder and samples above II. quality. Table 1 presents the data on the elemental composition across different quality grades of *A. bisporus* mushroom powder. It should be noted that the samples above II. Quality has been selected for the fortification of chocolate due to its significant protein content.

### 2.3. Chocolate Formulation and Sample Description

Three types of chocolate samples, dark chocolate, milk chocolate, and white chocolate, with varying percentages of mushroom powder, were developed and evaluated. This study assessed the total dietary fiber (TDF), protein content, mineral content, rheological properties, and sensory acceptability of enriched chocolate samples.

Hungarian Food Codex defines chocolate (dark) as Produced from cocoa products and sugar, contains at least 35% total cocoa dry matter, out of which there is at least 18% cocoa butter and at least 14% no-fat cocoa dry matter according to Codex Alimentarius Hungaricus 1-3-2000/36.

In our research, commercial dark chocolate (finest Belgian chocolate with 54.5%, 70.5% cocoa dry matter), milk chocolate (finest Belgian chocolate with 33.6% cocoa dry matter), and white chocolate (finest Belgian chocolate with 28% cocoa butter) were used to manufacture chocolate products with champignon mushroom powder.

Dark chocolate paste (54.5%) was made from 54.5 g/100 g of cocoa mass, 42.8 g of sugar, 5.1 g of protein; dark chocolate paste (70.5%) was made from 70.5 g/100 g of cocoa mass, 26 g of sugar, 8.8 g of protein; milk chocolate paste was made from 33.6 g/100 g of cocoa mass, 49.9 g of sugar, 7.0 g of protein; white chocolate paste was made from 28 g/100 g cocoa butter, 54.9 g of sugar, 6.0 g of protein mass. Cocoa butter with a fat content of 100 g/100 g was added to the samples, which required density adjustment. Control samples were made from dark chocolate, milk chocolate, and white chocolate mass without enrichment.

#### 2.3.1. Chocolate Tempering Process

This section describes the procedures used in this investigation. Each sample was prepared in the Food Innovation Center at the Institute of Food Technology, Faculty of Agricultural and Food Sciences, and Environmental Management, University of Debrecen, Hungary. Chocolate manufacturing processes typically involve the following steps: Mixing, Refining, Conching, Tempering, and Molding [2].

**Figure 2:**
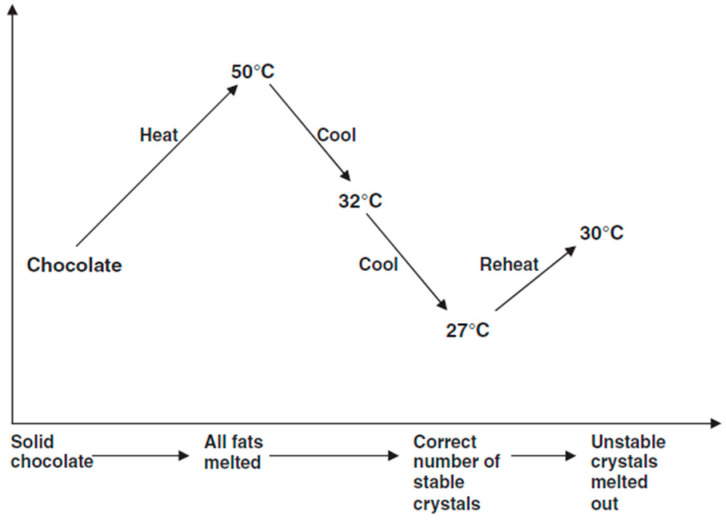
Tempering sequence during lipid crystallization in (dark) chocolate [2].

From Figure 2 we can see that tempering has four steps: melting to completion at 45 °C (in the case of white and milk chocolate), and 50 °C (in the case of dark chocolate), cooling to point of crystallization at 25 °C (in the case of white and milk chocolate), and 27 °C (in the case of dark chocolate), and conversion of any unstable crystals at 29 °C (in the case of white and milk chocolate), and 30–32 °C (in the case of dark chocolate) [42].

**Figure 3:**
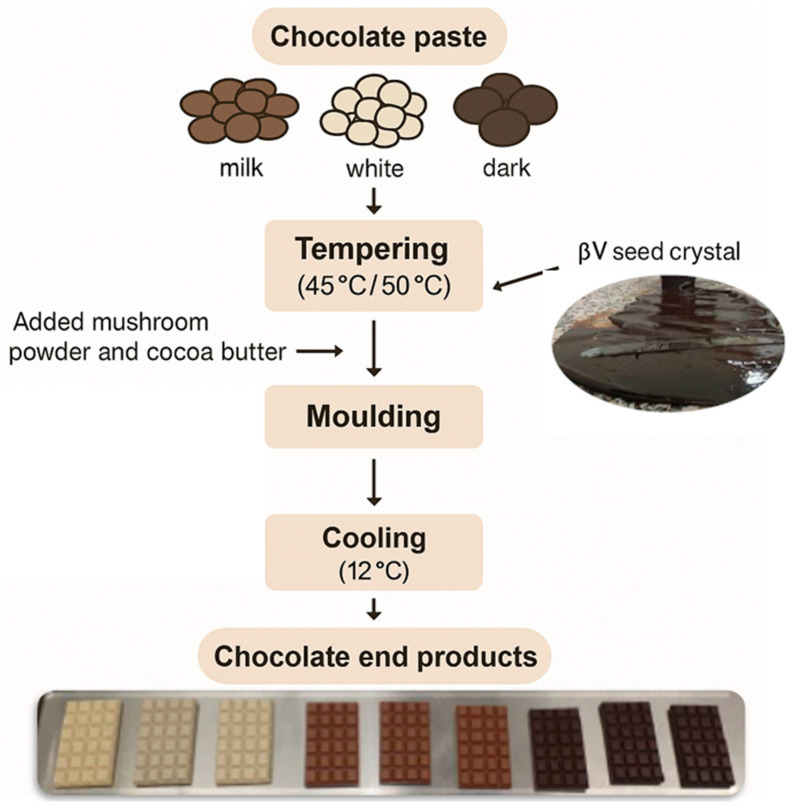
Chocolate Manufacturing Flow Chart (pictures were taken by Dr. Szintia Jevcsák at Food Innovation Center).

As shown in Figure 3, the chocolate manufacturing process starts with tempering. Cocoa butter can crystallize in several polymorphic forms, with fatty acid composition influencing how liquid fat solidifies [43]. Cocoa butter has from I to VI polymorphic forms, the principal being α, β, and β′. Form V is the most advisable, desirable form in well-tempered chocolate, which gives a glossy appearance, good snap, and resistance to blossom [44,45].

Our hand-tempering process is made in the Food Innovation Center. The following equipment has been used: a chocolate heating machine, a food thermometer, chocolate granite, chocolate spatulas, and a chocolate funnel.

#### 2.3.2. Sample Preparation for Analysis

**Figure 4:**
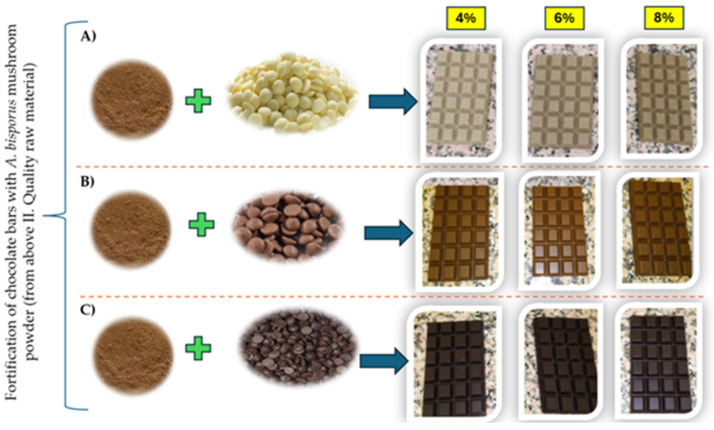
Fortified chocolate samples (with 4%, 6%, 8% mushroom powder from left), (**A**) Chocolate bars from white chocolate, (**B**) Chocolate bars from milk chocolate, (**C**) Chocolate bars from dark chocolate.

The choice of 4%, 6%, and 8% mushroom powder concentrations (Figure 4) was guided by earlier sensory evaluations, during which a more comprehensive range of concentrations was tested for consumer acceptability. These concentrations were selected based on a pre-experiment that assessed overall acceptability. Over 10% of the product had unpleasant sensory characteristics, and the texture was unacceptable. So, these particular levels (4%, 6%, and 8%) emerged as the most suitable, striking a balance between enhancing the chocolate’s functional properties—such as increasing bioactive compounds like polysaccharides, antioxidants, and dietary fiber—and minimizing any adverse sensory effects.

While higher concentrations could offer more excellent nutritional benefits, they risk amplifying the mushroom flavor or compromising the smooth texture of the chocolate, potentially reducing its appeal. The goal of selecting these moderate concentrations is to develop enriched milk chocolate varieties that meet diverse consumer preferences.

This strategy ensures that the added health benefits are achieved without compromising the sensory qualities essential for market success. This precise formulation reflects a commitment to innovation, seamlessly blending nutritional value with sensory satisfaction, and potentially setting a new standard in functional confectionery.

### 2.4. Measurements of the Mushroom Powder Fortified Samples

#### 2.4.1. Protein Content Analysis

The protein content of the samples was determined using the Kjeldahl method, in accordance with the MSZ EN 12135:1999 standard [46]. This classic technique, developed by Johann Kjeldahl in 1883, measures nitrogen in organic materials, which is then converted to protein using a factor. For chocolate, the factor used was 6.25. Each analysis was performed in triplicate for accuracy. The method includes three main steps: digestion, distillation, and titration. First, the sample is digested with concentrated sulfuric acid and catalyst tablets, breaking down organic matter and converting nitrogen into ammonium sulfate.

Then, sodium hydroxide is added, releasing ammonia during steam distillation, which is captured in boric acid. Finally, the ammonia and thus nitrogen is measured by titration using a color indicator. Exactly 1 g of chocolate was weighed to four decimal places on nitrogen-free paper, placed into a digestion tube, mixed with two catalyst tablets and 14 milliliters of sulfuric acid, then heated at 420 to 430 degrees Celsius for about two hours. A straightforward solution indicated complete digestion.

The required equipment included an analytical balance, digestion block, Kjeltec distillation unit, and Erlenmeyer flasks. Chemicals used were sulfuric acid, sodium hydroxide, boric acid, sodium carbonate, catalyst tablets (Kjeltabs S/3.5), and a suitable indicator.

#### 2.4.2. Dietary Fiber Determination

The total dietary fiber (TDF) content of the samples was determined using the enzymatic-gravimetric method, as described in Codex Alimentarius Hungaricus 3-2-2008/1. The digestion involved three enzymes: alpha-amylase, protease and amyloglucosidase. Fiber was precipitated from the samples, and the TDF filter bags were collected for analysis of ash and protein. The final fiber content was calculated using a standard formula.

To prepare the samples, fat and carbohydrates were first removed by drying. Then, three separate one-gram portions were each mixed with 40 milliliters of MES-TRIS buffer. Enzymatic hydrolysis is a three-step process. First, 50 microliters of heat-stable alpha-amylase was added and incubated in a water bath for 35 min. Next, 100 microliters of protease was added and held at 60 degrees Celsius for 30 min. After adjusting the pH to between 4.0 and 4.7, 300 microliters of amyloglucosidase was added, followed by another 30 min incubation at 60 degrees Celsius.

After enzyme treatment, the soluble fiber was precipitated using 95% ethanol at 60 °C. The solution was filtered through sintered glass crucibles lined with glass wool. The crucibles and residues were then dried at 105 °C, cooled in a desiccator, and weighed to determine the total dietary fiber content.

#### 2.4.3. Mineral Content Determination

The concentrations of the elements Al, B, Ba, Ca, Cr, Cu, Fe, K, Mg, Mn, Na, Ni, P, S, and Zn were determined by using an iCAP 6300 Dual (Thermo Electron Corporation, Waltham, MA, USA) Plasma Optical Emission Spectrometer (ICP-OES) [47]. The samples were prepared following the methodology outlined by Eriksen et al. (1996) [47]. In each experiment, 3 g of the sample were carefully measured into a 100 mL test tube. Subsequently, 10 mL of concentrated nitric acid was added to the samples, which were then allowed to rest overnight. This was followed by a 30 min heating process at 60 °C. Next, 3 mL of hydrogen peroxide was added to the samples, which were then heated for an additional 90 min at 120 °C. Then, the volume was adjusted to 50 mL using high-purity distilled water obtained from the Milli-Q water purification system (Millipore SAS, Molsheim, France). The samples were then filtered using 388 filter paper (Sartorius Stedim Biotech S.A., Gottingen, Germany).

Elemental analysis was conducted using an ICP-OES (Inductively Coupled Plasma Optical Emission Spectrometer) (Thermo Scientific iCAP 6300, Cambridge, UK), with measurements taken at specific wavelengths for each element: Ca (317.9 nm), K (766.4 nm), Mg (279.5 nm), Na (589.5 nm), P (185.9 nm), S (180.7 nm), Mn (259.3 nm), and Zn (213.8 nm). The ICP device operated at an output power of 1200 W.

#### 2.4.4. Texture Profile Analysis (TPA)

The textural characteristics of chocolate samples were evaluated instrumentally using a TA-XT Plus texture analyzer (Stable Micro Systems Ltd., Godalming, UK). Data analysis was calculated using a software package integrated into the instrument. The force-time curves provided valuable insights, enabling the determination of mechanical parameters, such as the firmness of samples [48,49]. Firmness was measured as the maximum peak force in grams per second (g/s). The texture parameters of this system have been estimated at room temperature [50,51].

#### 2.4.5. Sensorial Acceptability

The acceptability of food in sensory terms is influenced by its intrinsic properties, which are taste, appearance, and texture [52]. In our study, we assessed several of these sensory attributes, including taste, texture, appearance, flavor, the intensity of the mushroom note, the graininess of the texture, the overall harmony of ingredients, and overall liking. For this, we used a structured five-point hedonic scale, where 5 meant “liked very much” and 1 meant “disliked very much.

The sensory panel included 21 semi-trained tasters, young engineers, and PhD students from the university’s food science doctoral programs. Aged between 25 and 35, the group was almost evenly split by gender (11 women and 10 men). While they were not professional tasters, all were clearly interested in health-conscious eating and sustainable food solutions, making them a fitting group for evaluating mushroom-enriched products.

All tastings took place in a dedicated sensory lab under controlled conditions; neutral lighting, background, and other elements were consistent to reduce outside influences. Each chocolate sample was coded with a random three-digit number and served in a random sequence to avoid any bias from order effects. Panelists were also instructed to cleanse their palates with water between samples. The study was single-blind, meaning the tasters did not know the specific ingredients or formulation of the chocolates. To ensure freshness and consistency, all chocolate samples were vacuum-sealed and stored correctly, then brought to room temperature before being served.

### 2.5. Statistical Analysis

The tests were conducted in duplicates, and the results are presented as the mean values ± standard deviation (SD). Data analysis was performed using SPSS version 19.0 (Statistical Package for Social Sciences, Chicago, IL, USA). A one-way ANOVA was applied to compare the differences among group means, followed by post hoc Tukey’s multiple range test, with a significance level of *p* ≤ 0.05.

## 3. Results

### 3.1. Total Protein Content and Dietary Fiber Content of Chocolate Samples

**Figure 5:**
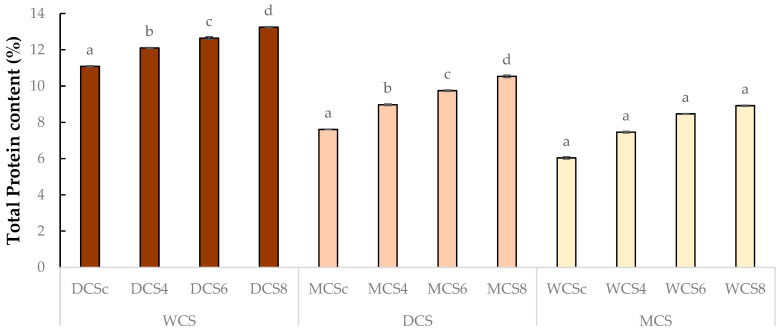
Total protein content of final products. WCS = white chocolate samples, DCS = dark chocolate samples, MCS = milk chocolate samples; WCSc, DCSc, MCSc = control samples; WCS4/6/8, DCS4/6/8, MCS4/6/8 = samples fortified with 4%, 6%, or 8% mushroom po-wder, respectively. Significant differences (*p* < 0.05) are described with different alphabets (a, b, c and d).

Table 2 and Figure 5. shows an increase in protein content in each sample fortified with white champignon mushroom powder. The white chocolate control sample had the lowest protein content, at 6.04% (*m*/*m*). The addition of 4%, 6%, and 8% mushroom powder increased the protein content from 7.46% to 8.92% (*m*/*m*). The highest level of protein content was observed in the dark chocolate (control sample with 11.09% (*m*/*m*)) fortified with 8% sous-vide white champignon mushroom powder, at 13.25% (*m*/*m*). Statistical analysis showed significant differences between groups in WCS (white chocolate samples). Significant differences were also determined between groups in DCS (dark chocolate samples).

**Figure 6:**
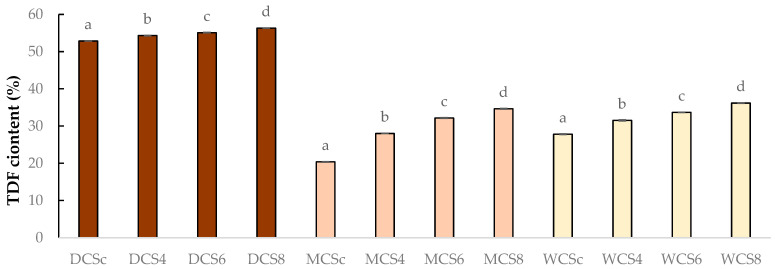
Total protein content of final products. WCS = white chocolate samples, DCS = dark chocolate samples, MCS = milk chocolate samples; WCSc, DCSc, MCSc = control samples; WCS4/6/8, DCS4/6/8, MCS4/6/8 = samples fortified with 4%, 6%, or 8% mushroom powder, respectively. Significant differences (*p* < 0.05) are described with different alphabets (a, b, c and d).

In MCS (milk chocolate samples), significant differences were not observed between groups. The results show that mushroom powder improves the nutritional value of chocolate products (Table 2). The results showed (Table 2 and Figure 6), that TDF content has increased according to the magnitude of mushroom powder enrichment. TDF content was 20.39%, 27.82%, and 52.86% in milk, white, and dark control samples. The dark chocolate fortified with 8% sous-vide white champignon mushroom powder had the highest TDF content, at 56.31%. Statistical analysis showed significant differences between each group.

### 3.2. Mineral Content of Chocolate Samples

Mushroom-enriched dark chocolate showed a high concentration in Mg, 2784 mg/kg was evaluated with 4% of enrichment, 3049 mg/kg was evaluated with 6%, and 3184 mg/kg was measured with 8% mushroom powder (the control sample showed 2579 mg/kg Mg concentration), and significant differences (*p* < 0.05) were observed amongst the samples. High concentrations of Ca were evaluated in white and milk chocolate samples, ranging from 3251 to 3826 mg/kg in WCS and from 2857 to 3565 mg/kg in MCS. Statistical analysis revealed significant differences between groups in terms of macroelements in WCS, except for Na. In addition, significant differences were observed between groups in DCS for each macroelement. In MCS, significant differences were determined between groups for Ca (Table 3).

Mushroom powder-enriched dark chocolate showed a high concentration in Fe; 116 mg/kg was determined in the control sample, and 119 to 135 mg/kg concentration was evaluated in enriched samples (116 mg/kg concentration was determined in the control sample). Significant differences (*p* < 0.05) were observed among the samples. Furthermore, high concentrations of Mn (22.3 to 28.2 mg/kg) and Zn (44.2 to 51.7 mg/kg) were also evaluated in dark chocolate samples. Mushroom powder significantly increased the Cu concentration in white chocolate products in the control sample, which was assessed at 3.25 mg/kg concentration, and in mushroom-enriched samples, was determined to be 18.06 mg/kg (WCS4), 19.04 mg/kg (WCS6), and 26.23 mg/kg (WCS8) concentration of Cu.

Statistical analysis revealed significant differences between groups in terms of microelements in WCS. Moreover, significant differences were observed between groups in DCS for each of the microelements. In MCS, significant differences were observed between groups for Al, B, Ba, and Cr (Table 4).

### 3.3. Results with Texture Profile

**Figure 7:**
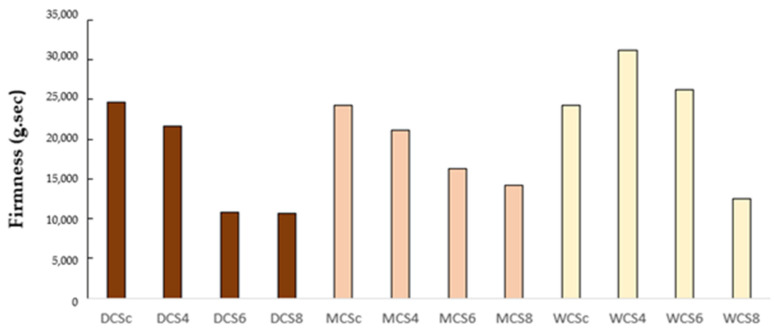
Texture properties of chocolate samples, g·s. Firmness is the maximum peak force in grams (g·s); resistance to chewing by the teeth. WCS = white chocolate samples, DCS = dark chocolate samples, MCS = milk chocolate samples. WCS = white chocolate samples, DCS = dark chocolate samples, MCS = milk chocolate samples; WCSc, DCSc, MCSc = control samples; WCS4/6/8, DCS4/6/8, MCS4/6/8 = samples fortified with 4%, 6%, or 8% mushroom powder, respectively.

The following section details the texture profile properties observed in chocolate samples fortified with mushroom powder. Firmness, measured as the maximum peak force (g·s) required to compress chocolate during chewing, was consistently lower in fortified samples compared to their respective control groups, as shown in Figure 7. This reduction was more pronounced as mushroom powder concentration increased across all chocolate types. Adding mushroom powder introduces bioactive compounds, including polysaccharides and fibers, which may affect the structural integrity of the chocolate matrix. These compounds could disrupt the crystalline and fat networks, resulting in a softer texture.

For instance, in the DCS8 formulation (8% mushroom powder), the firmness of dark chocolate decreased from 24,685 g·s in the control sample to 10,633 g·s, indicating a significant textural change.

Dark and milk chocolates followed a similar pattern of firmness reduction, but white chocolate exhibited an unusual trend, where WCS4 (31,229 g/s) had a higher firmness than the control sample (24,279 g/s). This anomaly may stem from variations in cocoa butter and sugar content, which could interact differently with mushroom-derived compounds. At a higher concentration (WCS8), the firmness aligned with the overall trend, dropping to 12,532 g·s.

### 3.4. Sensory Acceptability Profile

**Figure 8:**
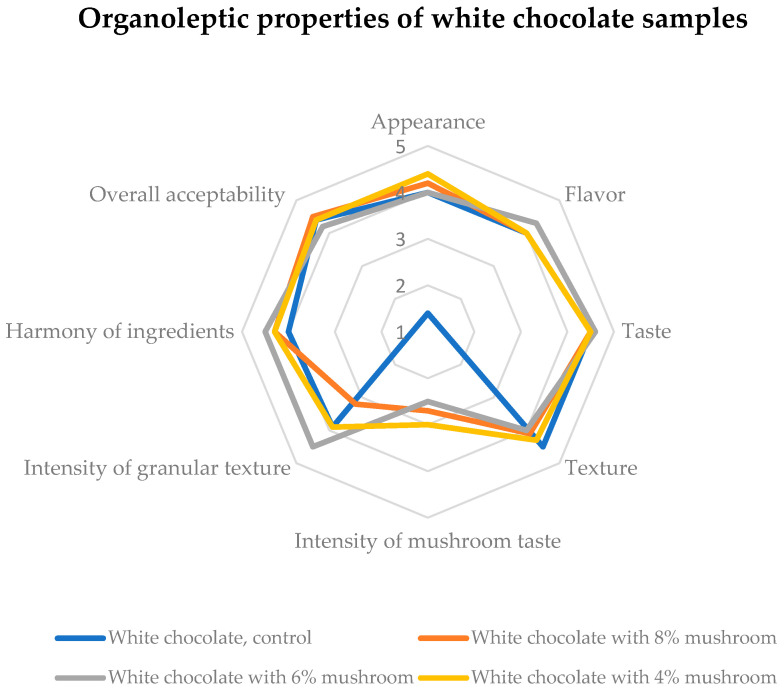
Sensory acceptability of white chocolate samples. Five-level evaluation scale for rating sample’s quality: 5 = very good, 4 = good, 3 = fair, 2 = poor, 1 = very poor.

Figure 8 presents the sensory profile of white chocolate samples, evaluated on a five-level scale (5 = very good, 4 = good, 3 = fair, 2 = poor, 1 = very poor). The data indicate that the enrichment process contributed to a “good” rating in critical attributes such as flavor, taste, harmony of ingredients, and overall acceptability. Our findings suggest that the integration of mushroom powder successfully maintained sensory quality while enhancing nutritional properties. However, future formulations may consider fine-tuning the enrichment level to achieve a higher rating, particularly for texture and aftertaste.

**Figure 9:**
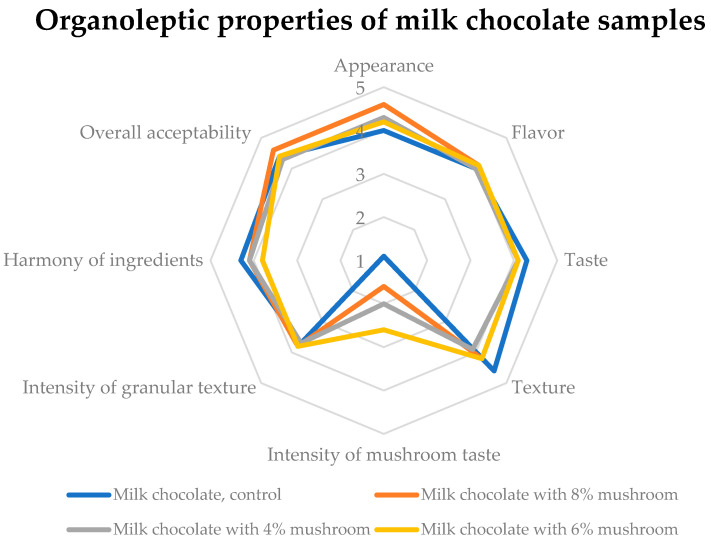
Sensory acceptability of milk chocolate samples. Five-level evaluation scale for rating sample’s quality: 5 = perfect, 4 = good, 3 = fair, 2 = poor, 1 = very poor.

Similarly, Figure 9 showcases the sensory acceptability of milk chocolate samples. The results reveal a “good” to “very good” evaluation for attributes like appearance and overall acceptability, underscoring the potential of mushroom powder as a functional ingredient. Nonetheless, it was noted that adding mushroom powder introduced a granular texture in some cases, which may detract from the sensory experience for specific consumers. To minimize this effect while preserving the overall sensory benefits, it is recommended that processing methods, such as finer milling of the mushroom powder or improved dispersion techniques, be explored.

**Figure 10:**
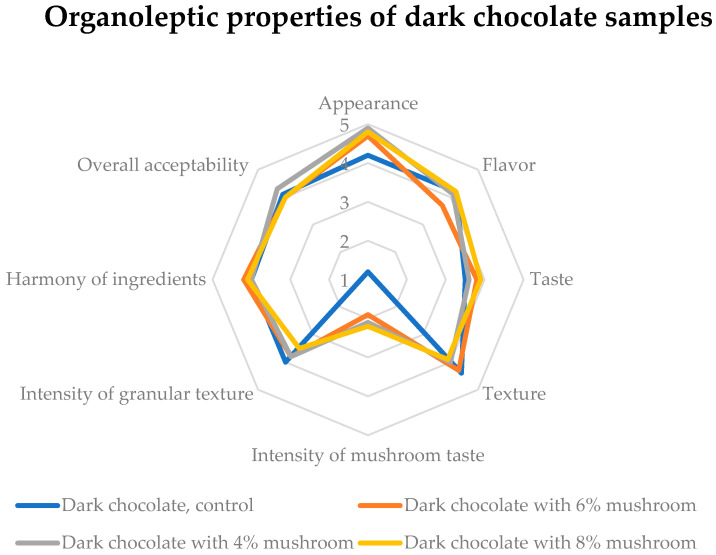
Sensory acceptability of dark chocolate samples. Five-level evaluation scale for rating sample’s quality: 5 = perfect, 4 = good, 3 = fair, 2 = poor, 1 = very poor.

Dark chocolate samples, including 6% and 8% mushroom powder, significantly enhanced the evaluation of appearance and taste, as depicted in Figure 10. The intensity of the mushroom flavor was subtle and non-disruptive, making it a suitable addition to dark chocolate formulations. To further optimize consumer acceptance, additional testing could assess the interaction between the mushroom powder and the chocolate matrix, particularly regarding mouthfeel and aftertaste. Variations in roasting or drying conditions for the mushroom powder could also be investigated to achieve an even smoother integration.

## 4. Challenges and Future Perspectives

Fortified chocolates with appealing taste and texture are emerging as an exciting innovation in the world of functional snacks. These enhanced treats are not only beneficial for health-conscious individuals but also hold promise for specific groups, such as children, the elderly, and those managing metabolic disorders. By offering a delicious and convenient format for nutrient delivery, chocolate fortification addresses the nutritional shortcomings of traditional chocolate, which is typically low in protein and fiber, transforming it into a more wholesome option that aligns with modern dietary trends and consumer expectations [53,54,55].

Various plant-based ingredients have already been explored for enriching chocolate, with spirulina standing out as a particularly effective option. This protein-rich microalga has shown significant results even in small amounts. Just 2% spirulina added to homemade chocolate dramatically boosts its protein content, outperforming many commercial baby foods [56].

Likewise, studies like that of Milagres et al. (2019) have shown that 70% cocoa chocolate naturally contains around 8.8% high-quality protein, which can be further enhanced through targeted enrichment strategies [57].

Among the latest innovations, mushrooms, especially *A. bisporus* (commonly known as white button mushrooms), offer a unique and promising approach to chocolate fortification. The current study demonstrated that integrating sous vide-processed mushroom powder into chocolate significantly improved its nutritional profile, especially in terms of protein, dietary fiber, and essential minerals. Dark chocolate, in particular, showed the greatest gains in nutritional density and enrichment efficiency. The best results came from the 8% mushroom-fortified dark chocolate, which reached a protein content of 13.25% and a dietary fiber content of 56.31%. Additionally, notable increases in magnesium, iron, and zinc reinforced the functional food potential of mushroom-enhanced chocolates.

Sensory evaluations supported the feasibility of this approach. Fortification with 6% and 8% mushroom powder was generally well received across dark, milk, and white chocolate types. Taste, appearance, and ingredient harmony all received positive feedback, especially for dark chocolate, where the mushroom’s mild earthy notes complemented the deep cocoa flavor. However, minor texture issues were observed, most notably in white chocolate, where firmness varied unexpectedly and a slightly gritty mouthfeel was noted.

Although chocolate enriched with *A. bisporus* mushroom powder offers nutritional and bioactive benefits, consumer acceptance may be limited by food neophobia. Some people might find the mix of mushrooms and chocolate unfamiliar or off-putting. Research shows that introducing new functional ingredients into familiar foods needs careful sensory testing and smart marketing to gain acceptance.

Further studies should explore flavor, texture, aroma, and overall appeal across different age and preference groups. Segmenting consumers by their openness to new foods and familiarity with functional products could help target the right audience. It is also worth testing how marketing and education can boost willingness to try such products. Lastly, dose–response studies should determine the ideal mushroom powder concentration that maintains health benefits without compromising taste.

Texture profile analysis revealed that higher mushroom content often resulted in softer textures across most samples. This is likely due to the disruptive effects of dietary fibers and polysaccharides on the chocolate’s fat and sugar structure [58]. While this resulted in a smoother, melt-in-the-mouth experience for dark and milk chocolates, improvements are still needed for white chocolate. Future efforts should focus on refining the powder’s particle size, improving dispersion methods, and optimizing formulations to reduce graininess and achieve a more uniform texture [59].

Despite these promising outcomes, challenges remain. A major concern is the stability of mushroom-derived bioactive compounds during processing, storage, and shelf life. Exposure to heat, oxygen, and interactions with lipids can degrade these valuable components, potentially diminishing their health benefits [60].

Additionally, the chocolate matrix itself is susceptible to oxidation and flavor changes over time, especially in products containing higher fiber or moisture-binding ingredients like mushroom powder. The presence of natural enzymes or residual moisture in the powder may further accelerate lipid oxidation or Maillard reactions, leading to undesirable off-flavors or discoloration [61,62,63]. Future studies must investigate the long-term chemical, sensory, and microbiological stability of mushroom-fortified chocolates under various storage conditions and packaging systems, such as vacuum-sealing or modified atmosphere packaging. add literature.

Moreover, scaling up production from the lab to industrial levels will require careful adjustment of processing parameters to ensure consistency, cost-efficiency, and consumer satisfaction [54].

A mushroom-fortified chocolate shows clear potential as a functional food, with both nutritional and sensory benefits already demonstrated. It can also serve as a natural and sustainable substitute for synthetic additives in chocolate fortification. However, for it to achieve mainstream acceptance and commercial success, several areas still need attention.

Looking ahead, future research should aim to validate health claims through clinical trials and long-term studies, particularly concerning metabolic health, immune support, and antioxidant effects [64]. It should also emphasize sustainability, highlighting the low environmental impact of mushroom cultivation and the potential to use byproducts in a circular economy model [65].

Broader consumer research is needed to confirm sensory acceptance and market viability. Additionally, cross-disciplinary innovation that combines food science, nutrition, and sustainability will be key to establishing mushroom-fortified chocolate as a credible functional food. By addressing these challenges, mushroom-enriched chocolates could evolve from promising experimental prototypes into scalable, sustainable, and widely accepted products in the growing functional confectionery market.

## 5. Conclusions

The development of chocolate enriched with sous vide-processed and freeze-dried white button mushroom (*Agaricus bisporus*) powder proved to be successful, resulting in a nutritionally enhanced product with increased protein, fiber, and mineral content. Among the tested variations, dark chocolate stood out with the highest nutritional value, while the 6% and 8% mushroom powder additions offered the best balance between improved health benefits, acceptable texture, and favorable taste. What makes this study unique is the use of gentle processing methods to produce high-quality mushroom powder, which is successfully incorporated into chocolate, one of the world’s most beloved treats. The final product retained its indulgent appeal while delivering notable nutritional upgrades, making it a thoughtful response to modern consumers’ growing demand for healthier snacks. Beyond its health benefits, the study also points to the sustainability advantages of mushrooms as fortifying agents. Mushroom cultivation is more efficient in terms of land, water, and energy use, especially when compared to animal-based protein production. Utilizing mushroom powders also supports circular economy principles by reducing waste and adding value to byproducts.

Future research should explore the long-term stability of these bioactive compounds in chocolate, test industrial-scale production, and evaluate both clinical outcomes and consumer acceptance. If these steps are realized, mushroom-fortified chocolate has the potential to move beyond the lab and establish itself as a credible, sustainable, and appealing addition to the functional food market.

## Figures and Tables

**Table 1:** Elemental Analysis of *Agaricus bisporus* Mushroom Powder (mg/kg for dry matter).

Tested Parameter	I. Quality	II. Quality	Above II. Quality
Al (mg/kg)	1.08 ± 0.25	0.34 ± 0.12	0.17 ± 0.02
B (mg/kg)	3.55 ± 2.05	4.17 ± 0.34	1.25 ± 0.41
Ba (mg/kg)	0.12 ± 0.00	-	-
Ca (mg/kg)	71.82 ± 33.67	64.64 ± 1.88	62.84 ± 9.22
Cr (mg/kg)	0.18 ± 0.00	0.20 ± 0.02	0.18 ± 0.01
Cu (mg/kg)	1.26 ± 0.47	0.79 ± 0.40	0.15 ± 0.40
Fe (mg/kg)	2.54 ± 0.57	2.06 ± 0.37	2.46 ± 0.27
K (mg/kg)	2518.25 ± 82.76	2620.00 ± 307.41	2604.75 ± 407.51

**Table 2:** Protein and Total dietary fiber content of chocolate samples % (*m*/*m* for dry matter).

Samples	TP Content % (*m*/*m*)	TDF Content (%)
DCSc	11.09 ± 0.03 ^d^	52.86 ± 0.07 ^d^
DCS4	12.10 ± 0.03 ^c^	54.32 ± 0.04 ^c^
DCS6	12.65 ± 0.07 ^b^	55.09 ± 0.09 ^b^
DCS8	13.25 ± 0.03 ^a^	56.31 ± 0.11 ^a^
MCSc	7.61 ± 0.01 ^a^	20.39 ± 0.07 ^d^
MCS4	8.97 ± 0.04 ^a^	28.02 ± 0.13 ^c^
MCS6	9.75 ± 0.04 ^a^	32.17 ± 0.03 ^b^
MCS8	10.54 ± 0.06 ^a^	34.65 ± 0.08 ^a^
WCSc	6.04 ± 0.06 ^d^	27.82 ± 0.05 ^d^
WCS4	7.46 ± 0.04 ^c^	31.53 ± 0.07 ^c^
WCS6	8.47 ± 0.01 ^b^	33.69 ± 0.07 ^b^
WCS8	8.92 ± 0.03 ^a^	36.19 ± 0.03 ^a^

Results are reported as fresh weight and expressed as the mean values ± standard deviations for three replications. Values with different letters indicate significant differences determined by Tukey’s test (*p* < 0.05). WCS = white chocolate samples, DCS = dark chocolate samples, MCS = milk chocolate samples; WCSc, DCSc, MCSc = control samples; WCS4/6/8, DCS4/6/8, MCS4/6/8 = samples fortified with 4%, 6%, or 8% mushroom powder, respectively.

**Table 3:** Macroelements of chocolate samples for dry matter in mg/kg.

Samples	Ca	K	Mg	Na	P	S
**DCSc**	1464 ± 9.93 ^d^	6673 ± 10.89 ^d^	2579 ± 8.24 ^d^	208 ± 1.69 ^c^	3667 ± 5.74 ^d^	1302 ± 1.64 ^d^
**DCS4**	1605 ± 5.63 ^c^	8175 ± 20.44 ^c^	2784 ± 7.63 ^c^	250 ± 0.91 ^b^	3711 ± 4.92 ^c^	1376 ± 7.38 ^c^
**DCS6**	1784 ± 10.14 ^b^	8717 ± 7.40 ^b^	3049 ± 18.50 ^b^	254 ± 2.97 ^b^	3949 ± 8.40 ^b^	1444 ± 7.33 ^b^
**DCS8**	2011 ± 2.00 ^a^	8951 ± 6.67 ^a^	3184 ± 5.60 ^a^	277 ± 3.00 ^a^	4120 ± 12.10 ^a^	1773 ± 10.05 ^a^
**MCSc**	2857 ± 9.44 ^d^	3794 ± 2.90 ^a^	788 ± 6.40 ^a^	383 ± 2.19 ^a^	2294 ± 7.37 ^a^	857 ± 4.53 ^a^
**MCS4**	3257 ± 11.55 ^c^	4769 ± 2.92 ^a^	841 ± 5.81 ^a^	428 ± 1.11 ^a^	2648 ± 4.24 ^a^	926 ± 5.47 ^a^
**MCS6**	3565 ± 9.60 ^b^	5138 ± 10.04 ^a^	941 ± 4.20 ^a^	556 ± 2.36 ^a^	2701 ± 5.45 ^a^	990 ± 6.64 ^a^
**MCS8**	2603 ± 5.52 ^a^	5752 ± 1.21 ^a^	975 ± 1.82 ^a^	580 ± 0.92 ^a^	2982 ± 0.94 ^a^	1085 ± 4.41 ^a^
**WCSc**	3251 ± 1.70 ^d^	2948 ± 21.67 ^d^	425 ± 8.59 ^c^	388.8 ± 0.27 ^a^	2089 ± 3.29 ^d^	796 ± 5.55 ^d^
**WCS4**	3379 ± 13.75 ^c^	3171 ± 19.90 ^c^	453 ± 0.49 ^b^	453.5 ± 2.33 ^a^	2392 ± 8.88 ^c^	816 ± 2.98 ^c^
**WCS6**	3693 ± 13.11 ^b^	3363 ± 8.51 ^b^	477 ± 13.96 ^b^	471.5 ± 12.91 ^a^	2490 ± 2.46 ^b^	859 ± 2.38 ^b^
**WCS8**	3826 ± 8.18 ^a^	4378 ± 5.35 ^a^	566 ± 4.73 ^a^	605.4 ± 2.84 ^a^	2724 ± 7.67 ^a^	912 ± 4.27 ^a^

Results are reported as fresh weight and expressed as the mean values ± standard deviations for two replications. Values with different letters indicate significant differences determined by Tukey’s test (*p* < 0.05). WCS = white chocolate samples, DCS = dark chocolate samples, MCS = milk chocolate samples; WCSc, DCSc, MCSc = control samples; WCS4/6/8, DCS4/6/8, MCS4/6/8 = samples fortified with 4%, 6%, or 8% mushroom po-wder, respectively.

**Table 4:** Microelements of chocolate samples, mg/kg.

Samples	Al	B	Ba	Cr	Cu	Fe	Mn	Ni	Zn
**DCSc**	43.0 ± 0.20 ^d^	14.6 ± 0.02 ^d^	6.07 ± 0.08 ^c^	1.010 ± 0.01 ^d^	24.1 ± 0.04 ^d^	116 ± 0.18 ^d^	22.3 ± 0.11 ^d^	4.55 ± 0.18 ^b^	44.2 ± 0.34 ^d^
**DCS4**	49.5 ± 0.07 ^c^	15.9 ± 0.05 ^c^	6.61 ± 0.02 ^b^	1.160 ± 0.01 ^c^	26.1 ± 0.02 ^c^	119 ± 0.63 ^c^	24.2 ± 0.09 ^c^	4.59 ± 0.05 ^b^	45.4 ± 0.06 ^c^
**DCS6**	55.8 ± 0.29 ^b^	16.3 ± 0.02 ^b^	6.70 ± 0.01 ^b^	1.290 ± 0.02 ^b^	26.7 ± 0.03 ^b^	126 ± 0.13 ^b^	25.3 ± 0.42 ^b^	5.03 ± 0.08 ^a^	50.5 ± 0.13 ^b^
**DCS8**	64.7 ± 0.02 ^a^	17.4 ± 0.19 ^a^	7.36 ± 0.05 ^a^	1.510 ± 0.03 ^a^	28.0 ± 0.03 ^a^	135 ± 0.50 ^a^	28.2 ± 0.05 ^a^	5.13 ± 0.02 ^a^	51.7 ± 0.27 ^a^
**WCSc**	34.5 ± 0.03 ^d^	5.04 ± 0.00 ^d^	1.48 ± 0.00 ^c^	0.806 ± 0.01 ^c^	3.25 ± 0.04 ^d^	26.1 ± 0.05 ^d^	1.12 ± 0.03 ^c^	0.363 ± 0.01 ^d^	14.3 ± 0.07 ^d^
**WCS4**	36.0 ± 0.03 ^c^	5.83 ± 0.02 ^c^	1.50 ± 0.02 ^c^	0.988 ± 0.02 ^b^	18.06 ± 0.02 ^c^	29.3 ± 0.07 ^c^	1.38 ± 0.03 ^b^	0.466 ± 0.01 ^c^	21.5 ± 0.03 ^c^
**WCS6**	38.3 ± 0.07 ^b^	7.22 ± 0.09 ^b^	2.04 ± 0.03 ^b^	0.995 ± 0.01 ^b^	19.04 ± 0.03 ^b^	32.4 ± 0.02 ^b^	1.39 ± 0.02 ^b^	0.510 ± 0.01 ^b^	22.3 ± 0.09 ^b^
**WCS8**	49.8 ± 0.03 ^a^	8.17 ± 0.01 ^a^	2.67 ± 0.05 ^a^	1.077 ± 0.02 ^a^	26.23 ± 0.12 ^a^	36.5 ± 0.04 ^a^	1.52 ± 0.02 ^a^	0.543 ± 0.00 ^a^	24.9 ± 0.03 ^a^
**MCSc**	24.4 ± 0.12 ^d^	5.20 ± 0.03 ^d^	1.49 ± 0.01 ^d^	0.526 ± 0.01 ^c^	5.29 ± 0.01 ^a^	31.7 ± 0.01 ^b^	4.24 ± 0.08 ^a^	0.571 ± 0.01 ^b^	18.4 ± 0.09 ^a^
**MCS4**	28.7 ± 0.07 ^c^	6.33 ± 0.04 ^c^	2.03 ± 0.16 ^c^	0.534 ± 0.01 ^c^	8.46 ± 0.02 ^a^	51.2 ± 0.05 ^b^	4.41 ± 0.01 ^a^	0.808 ± 0.02 ^b^	19.3 ± 0.13 ^a^
**MCS6**	33.0 ± 0.10 ^b^	7.44 ± 0.01 ^b^	2.61 ± 0.01 ^b^	0.704 ± 0.02 ^b^	9.44 ± 0.05 ^a^	68.6 ± 0.62 ^b^	5.07 ± 0.05 ^a^	1.00 ± 0.08 ^a^	27.7 ± 0.30 ^a^
**MCS8**	47.5 ± 0.02 ^a^	8.93 ± 0.02 ^a^	2.86 ± 0.03 ^a^	0.867 ± 0.01 ^a^	10.60 ± 0.01 ^a^	119 ± 0.15 ^a^	5.48 ± 0.03 ^a^	1.19 ± 0.01 ^a^	31.7 ± 0.08 ^a^

Results are reported as fresh weight and expressed as the mean values ± standard deviations for two replications. Values with different letters indicate significant differences determined by Tukey’s test (*p* < 0.05). WCS = white chocolate samples, DCS = dark chocolate samples, MCS = milk chocolate samples; WCSc, DCSc, MCSc = control samples; WCS4/6/8, DCS4/6/8, MCS4/6/8 = samples fortified with 4%, 6%, or 8% mushroom po-wder, respectively.

## Data Availability

The original contributions presented in this study are included in the article. Further inquiries can be directed to the corresponding authors.
